# Mandrake: multiagent systems as a basis for programming fault-tolerant decentralized applications

**DOI:** 10.1007/s10458-021-09540-8

**Published:** 2022-02-08

**Authors:** Samuel H. Christie, Amit K. Chopra, Munindar P. Singh

**Affiliations:** 1 Lancaster University, Lancaster, UK; 2 North Carolina State University, Raleigh, NC 27695 USA

**Keywords:** Fault tolerance, Agent programming, Protocols

## Abstract

We conceptualize a *decentralized* software application as one constituted from *autonomous* agents that communicate via *asynchronous* messaging. Modern software paradigms such as microservices and settings such as the Internet of Things evidence a growing interest in decentralized applications. Constructing a decentralized application involves designing agents as independent local computations that coordinate successfully to realize the application’s requirements. Moreover, a decentralized application is susceptible to faults manifested as message loss, delay, and reordering. We contribute *Mandrake*, a programming model for decentralized applications that tackles these challenges without relying on infrastructure guarantees. Specifically, we adopt the construct of an *information protocol* that specifies messaging between agents purely in causal terms and can be correctly enacted by agents in a shared-nothing environment over nothing more than unreliable, unordered transport. Mandrake facilitates (1) implementing protocol-compliant agents by introducing a programming model; (2) transforming protocols into fault-tolerant ones with simple annotations; and (3) a declarative policy language that makes it easy to implement fault-tolerance in agents based on the capabilities in protocols. Mandrake’s significance lies in demonstrating a straightforward approach for constructing decentralized applications without relying on coordination mechanisms in the infrastructure, thus achieving some of the goals of the founders of networked computing from the 1970s.

## Introduction

We conceptualize a *decentralized* software application as one that involves two or more *autonomous* endpoints, or *agents*. We assume a communication infrastructure based on *asynchronous* messaging since that reduces coupling between the agents. Although our approach could be realized on more restrictive infrastructures, for generality, we focus on asynchrony without ordering guarantees.

We observe two motivations for decentralization. First, decentralization reflects autonomy in the overarching social architecture of an application [21]. Applications in domains such as e-commerce, finance, and healthcare span multiple autonomous real-world parties. Second, decentralization reflects loose coupling in the technical architecture. The *microservices* paradigm [41] supports developing, deploying, and scaling microservices independently of each other. The Internet of Things (IoT) motivates decentralization in both social and technical terms [36, 54] by bringing forth interactions between devices owned by two or more parties and by technologies such as fog computing that distribute information processing and storage [43].

However, getting decentralized applications right is extremely difficult. Asynchrony and faults make coordinating the computations of an application challenging. This challenge is exacerbated when, as in open applications, agents represent autonomous real-world parties and are independently constructed. Further, autonomy motivates flexibility in interactions [58]; however, flexibility itself is in tension with ease of coordination [8, 56].

In conventional approaches for building distributed systems, coordination, including consistency and fault tolerance, is addressed in an application’s communication infrastructure via guarantees for reliable and ordered delivery of messages. TCP, e.g., implements complex reliability and ordering mechanisms. Already there is a move away from TCP in several domains for performance reasons. E.g., in the IoT, lightweight communication services such as CoAP [50] are preferable to TCP-based protocols such as HTTP and AMQP [1]. Performance though is not the only reason move away from complex communication services. There is a fundamental systems principle, the end-to-end model [47], that argues against reliability and ordering guarantees fixed in the infrastructure. The argument is twofold. One, such guarantees turn out to be redundant considering what must be implemented at the application level to make an application robust. Two, and worse, they interfere with *application meaning* [19], which lies in the application domain and refers to how users make distributed decisions in practice. Specifically, infrastructure guarantees hamper flexibility by preventing agents from observing some sequences of events (and thus computations) that are legitimate from the application perspective and that agents could benefit from in practice. Traditional fault tolerance, in particular, is not meaningful, because it is transparent to the application and neither influences nor considers agent decisions. Naturally, a communication service that has redundant features and interferes with application reasoning is likely to result in poorer performance than if concerns of consistency and fault tolerance were left to the application.

### Contributions

This paper addresses the challenges of building robust decentralized applications in a manner compatible with the end-to-end model. In particular we contribute *Mandrake*, a set of techniques that demonstrates how a fault-tolerant decentralized application can be realized as a multiagent system sitting on top of an infrastructure that provides neither ordering nor reliability guarantees. Mandrake is based on the insight that to support meaning, a decentralized application must be modeled via a declarative information protocol [52]. An information protocol captures the interactive part of application meaning by constraining an agent’s emission of messages based purely on its local information state, which is essentially the agent’s history of communications. Receptions, by contrast, are unconstrained. Specifically, an agent may receive messages sent by others in any order. An agent’s decision making (which would generally rely both on its local state and internal state) is up to the agent’s implementation. Taken together, the possibility of an agent receiving messages in any order and processing them in accordance with its own decision making are crucial to realizing application meaning.

Such an application architecture provides an opportunity to introduce meaningful fault tolerance—that is, fault tolerance based on information available to agents and incorporated in their decision making. In particular, it enables understanding a fault as the violation of an *expectation* of an agent to receive some information from others and fault tolerance as what agents do to prevent or handle the violations.

Concretely, Mandrake makes the following technical contributions.

- A transformation language for extending protocols with common communication patterns that enable fault tolerance, such as forwarding information.
- A high-level programming model that enables realizing agents that enact information protocols. The programming model motivates a specific agent architecture and is realized via a concrete API for implementing agents.
- A declarative language for specifying fault tolerance policies, to simplify the development of robust, application-specific agents.

Mandrake builds upon recent contributions that take advantage of information protocols to enable application-level fault tolerance. Fault tolerance based on information protocols was first proposed in [22]. Christie et al. [24] demonstrate that information protocols enable constructing agents that can recover from message loss; however, the programming model support for constructing such agents was limited to message validation. It provides neither an API nor a language for specifying fault tolerance policies. Bungie [23] informally presents fault-tolerance patterns that can be applied to information protocols to obtain more robust protocols. Mandrake formalizes those intuitions in a transformation language.

### Organization of the paper

Section 2 describes a health care scenario, and gives a protocol specification. Section 3 describes the need for fault tolerance, specifically at the application level, and describes the annotations we introduce for transforming protocols to support fault tolerance. Section 4 describes our proposed programming model, including architecture, agent programming API, and a declarative fault tolerance policy specification language. Section 5 gives a conceptual evaluation and comparison with the Jason agent programming framework. Section 6 contrasts Mandrake with related work in the areas of fault tolerance, services, and MAS development. Section 7 summarizes our conclusions and identifies several directions for future work.

## Scenario specification

Our running example is a simple medical treatment scenario: The patient may send a complaint to the doctor describing the symptoms they have been experiencing. In response, the doctor may send a prescription to a pharmacist, who fills the prescription for the patient.

Listing 1 gives a specification of a basic prescription process at the interaction level written in the information-based protocol language BSPL [52]. 
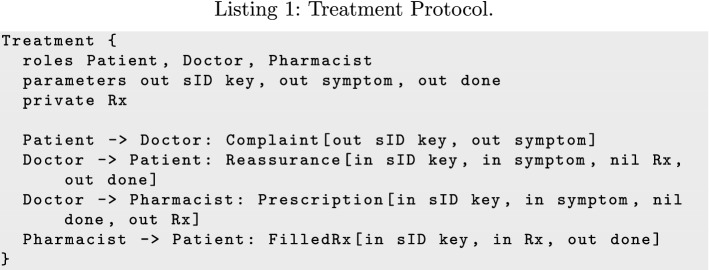


An information protocol specifies an interaction in terms of messages between roles, the information those messages carry, and causality and integrity constraints on those information parameters.

The *Treatment* protocol involves three roles (Patient, Doctor, and Pharmacist) and consists of four messages between these roles. Each message has a payload of parameters that can be adorned $\ulcorner \mathsf {in}\urcorner$, $\ulcorner \mathsf {out}\urcorner$, or $\ulcorner \mathsf {nil}\urcorner$, and of which at least one must be a $\mathsf {key}$. These parameter adornments define causality constraints on the basis of whether or not a binding for that parameter is known. Parameter bindings occur within the context of an enactment identified by the bindings of $\mathsf {key}$ parameters; within an enactment, each parameter may be bound only once for integrity. All constraints are on the emission of messages; message reception is unconstrained except that a message must be emitted to be received.

Parameters adorned $\ulcorner \mathsf {out}\urcorner$ create new bindings and so must not already have a binding when the message is sent; for example, Patient creates a new binding for symptom when they send *Complaint*. Parameters adorned $\ulcorner \mathsf {in}\urcorner$ identify dependencies, and their binding must be known before the message can be sent; for example, Doctor cannot send *Prescription* until they know the binding of symptom. Finally, $\ulcorner \mathsf {nil}\urcorner$ parameters *prevent* a message from being sent if a binding is known; for example, the $\ulcorner \mathsf {nil}\urcorner$ Rx in *Reassurance* prevents Doctor from sending it if they have already sent *Prescription* (which binds Rx).

Patient can begin an enactment of *Treatment* by sending *Complaint*, which has no $\ulcorner \mathsf {in}\urcorner$ parameters and thus no dependencies. Sending *Complaint* introduces new bindings for the sID and symptom parameters because they are adorned $\ulcorner \mathsf {out}\urcorner$. Once Doctor observes the binding for sID and symptom, they have enough information to either send *Reassurance* or *Prescription*. Doctor chooses between *Reassurance* and *Prescription*: *Reassurance* binds done which blocks *Prescription*, and *Prescription* binds Rx which blocks *Reassurance*. A protocol enactment is complete when all its public $\ulcorner \mathsf {out}\urcorner$ parameters are bound, so *Reassurance* completes the enactment of *Treatment* when it binds done. If Doctor chooses to send *Prescription* instead, the enactment is completed when Pharmacist sends *FilledRx* and binds done.

Correlation based on key parameters makes duplicated messages idempotent; only the first reception results in a new observation. Correlation is the basis for handling multiple complaints concurrently; each *Complaint* message has its own sID, and is thus either a duplicate (and ignored) or the beginning of a distinct interaction. Importantly, key-based correlation makes information protocols robust against message reordering, since messages are interpreted by their contents instead of their order.

Key parameters model semantic identifiers, that is, meaningful identifiers for correlation that are part of the application domain. The use of semantic identifiers for correlation and idempotence is not novel, but it is commonplace to rely on arbitrary syntactic identifiers instead—for example, AMQP’s correlation ID is a single parameter external to the message (and thus not meaningful within the interaction) for correlating request/response pairs.[20]. De Graauw [29], e.g., notes the limitations of using reliable messaging protocols for Web services and advocates using semantic identifiers for idempotence and fault tolerance. BSPL provides language features for modeling semantic identifiers, enabling programming models such as Mandrake that can exploit them for correlation.^1^

## Fault tolerance

Under ideal conditions, the above protocol sufficiently specifies the system: each participant is enabled to perform the actions necessary for filling the prescription, yet constrained from doing things they shouldn’t, such as producing inconsistent bindings.

However, if any of the messages is lost, or if an agent does not perform an expected task (e.g., doctor fails to send the prescription), then the prescription will not be filled. We want to *ensure* that the patient will eventually get their medication, even if some things go wrong.

This concept of maintaining correctness and progress despite faults is *fault tolerance*. However, most work on fault tolerance, which we shall call *infrastructure-level fault tolerance*, has been focused on addressing the sources of faults rather than achieving the desired outcome. This can be seen from the cause-based perspective of the most common fault tolerance taxonomies [6]. The resulting methods are independent of the application, and either focused on specific errors (e.g., memory faults) or generic solutions (e.g., redundant components with automatic failover). Instead, we focus on *application-level fault tolerance*, using higher-level concepts to work directly toward the desired outcome.

Take Patient’s submission of a complaint for example. An infrastructure-level approach might be to use TCP, which automatically resends packets until they are acknowledged to recover from packet loss during transmission. However, infrastructure-level acknowledgments are basically meaningless, because they are not produced intentionally by the agent. Such acknowledgments cover the smallest possible amount of progress, mere transmission, without verifying that the agent has received, accepted, or processed the messages.

### Application-level fault tolerance

An application-level approach should both reassure that progress is being made and help the agent recover from problems. For example, the doctor could forward a copy of the *Prescription* to the patient. The forwarded prescription both proves that progress has been made, and enables new recovery options for the patient, as the patient can now forward the *Prescription* directly to the pharmacist. If all goes well the agents directly interact with each other for maximum efficiency, but if something goes wrong the patient knows where and how to resume the enactment.

Meaningful communication is helpful for fault tolerance, because it supports and informs agent expectations based on the protocol. After submitting a complaint, Patient expects to receive either reassurance or a filled prescription. If this expectation is not met within a reasonable time (as determined by Patient), Patient may assume that something has failed and attempt recovery, perhaps by resending their *Complaint* to the doctor, or by more directly pursuing the fulfillment of their prescription and contacting the pharmacist.

### Approach overview

After identifying the completion requirements and agent expectations from the protocol, we propose using the following communication patterns to add robustness to a MAS and support fault tolerance:

1. (*Remind*) Retry sending messages to recover from failed expectations;
  e.g., remind Doctor of *Complaint*.
2. (*Checkpoint*) Forward intermediate information to an agent with expectations, so they are aware of progress;
  e.g., forward *Prescription* to Patient.
3. (*Continue*) Forward intermediate information to continue from checkpoint, instead of redoing work;
  e.g., forward *Prescription* from Patient to Pharmacist.

These patterns are implemented by adding messages to the protocol, to capture the additional actions that can be taken. Although these changes could be made directly to the protocol, we propose the use of syntactic transformations applied to the protocol. Such transformations enable capturing the higher-level semantics of the change being made, and reduce the effort required for implementing these patterns by abstracting them. Thus, transformations represent a middle-ground compromise between directly modifying the existing protocol (fully backwards-compatible, but loses information about the transformation made), and developing a new protocol language that incorporates concepts such as retries or forwarding directly (which sacrifices backwards compatibility, both at the implementation and theoretical levels).

The following subsections show how to transform the protocol to support these patterns, and implement agent policies to use them.

### Transforming protocols via annotations

To support fault tolerance, we can extend the protocol to include additional messages that enable the agents to make progress despite faults. These additional messages are working toward existing objectives and thus ultimately communicate the same information, so they can be derived from existing messages according to common patterns.

Although we could take advantage of the idempotence of information-based protocols and resend the original messages, we instead construct new messages for two reasons: First, a new message conveys the information that there is an attempted recovery. The message may introduce a new key parameter to permit multiple distinguishable recovery attempts. Second, new messages can add completely new communication patterns, such as having the Patient forward a copy of their prescription directly to Pharmacist instead of asking Doctor to resend the prescription.

To reduce the effort of adding messages to a protocol, we introduce the concept of tooling-supported protocol *transformations*. A transformation takes a protocol and adds messages according to the designer’s specifications. In the following listings, we give examples of transformations applied to messages from *Treatment*, followed by the output each transformation produces.

Our primary protocol transformation pattern is the *forward*, which resends some information from one agent to another; possibly though not necessarily between the original agents. The forward transformation is the basis for all three of our patterns mentioned in Sect. 3.2. For example, the Patient can remind Doctor about their symptoms by forwarding the *Complaint* if they don’t receive any treatment. We forward messages in our protocol using another message containing all the same parameters plus a new key to identify the instances of forwarding. 
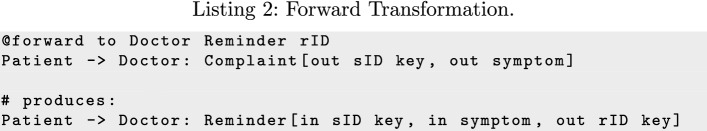


Listing 2 shows the @forward transformation. The annotation has three arguments: the recipient to forward the message to, the name to use for the new message, and a new key parameter. To support multiple forwards relaying the same message, the annotation can also take a from argument. Expanding this reminder transformation produces the *Reminder* message, which contains the same parameters as the original *Complaint* message, plus the new key.

For some patterns we provide additional shorthand. For example, as Listing 3 shows, to support the *Remind* pattern, @remind is shorthand for forwarding a message from the original sender to the original recipient. 
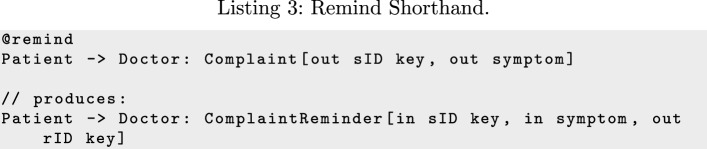


Note that transformation can generate message and key names if they are not provided.

The examples of forwarding in Listings 2 and 3 are direct, between the original sender and recipient. In larger multiparty protocols, however, indirect reminders can greatly increase the range of possible fault recovery actions. By *indirect* reminders, we mean reminders received from an agent other than the original sender. Indirect reminders are used in the *Checkpoint* and *Continue* patterns. For example, the Doctor may give Patient a copy of the prescription (*Checkpoint*), to enable Patient to remind Pharmacist about the prescription directly (*Continue*). Indirect reminders are simple but powerful concepts for application-specific fault tolerance, endowing parties with necessary local information to directly respond to faults or follow up on incomplete tasks.

We further support indirect reminders through two additional transformations, route and gossip: 
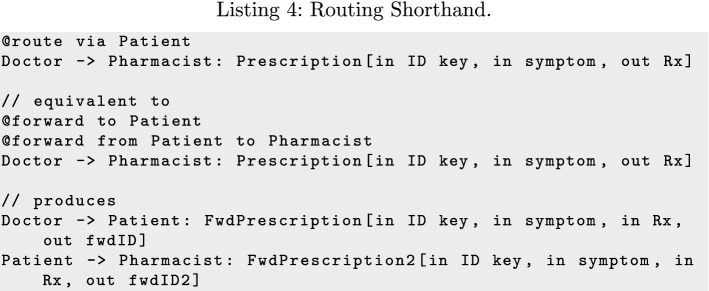


The @route annotation takes as parameters a sequence of intermediary agents through which to route the forwarding of a message, and expands to a sequence of forwards. In the example in Listing 4, @route is used to generate messages enabling Doctor to forward the prescription to Patient, who can then forward it again to Pharmacist. 
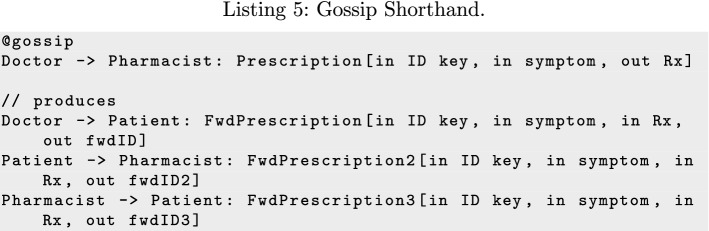


Similarly, the @gossip transformation generates a complete transmission graph among a set of roles (defaulting to all roles), so each peer can forward a message to its neighbors to eventually reach everyone. Gossip is distinct from the concept of broadcast, which has the source directly transmit to all recipients. Listing 5 shows how applying the @gossip transformation to the *Prescription* message adds new forwarding messages to the protocol so that any of the agents can forward it to the others. The gossip transformation enables the agents to enact any propagation policy they choose, and can be an effective means of transmitting messages if the topology of the network changes over time (as it would with agent failure) [48].

## Programming model

**Fig. 1:**
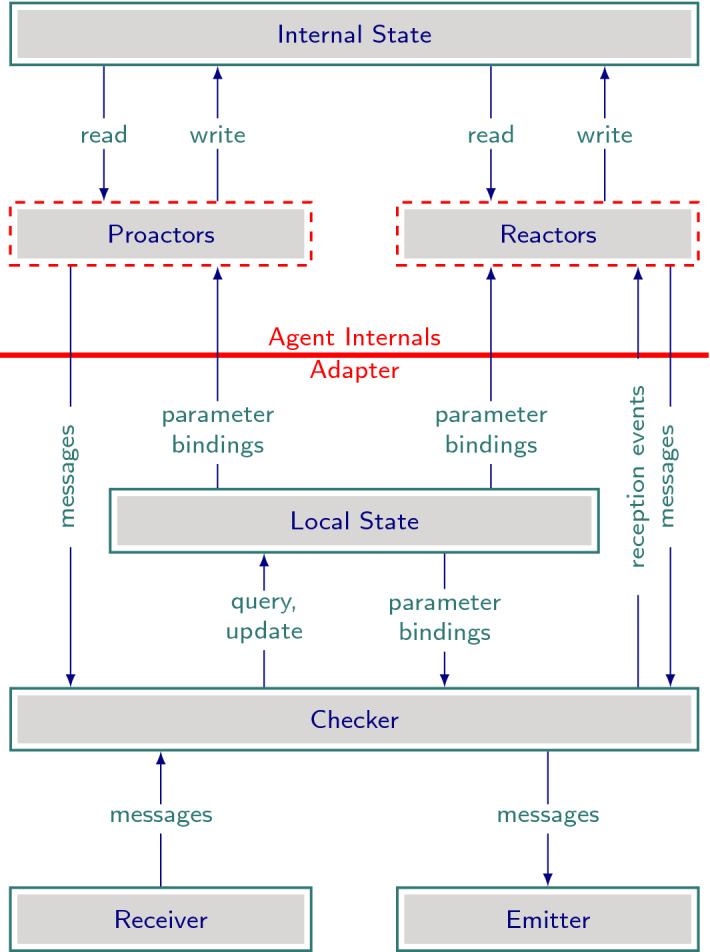
Agent architecture

Figure 1 shows the architecture of a Mandrake agent. Each role in a protocol has a logical *skeleton* consisting of the messages it is enabled to send and receive; this skeleton is provided by the adapter components below the center line. An operational agent fleshes out this skeleton with internal business logic that drive its interactions with other agents by determining when to send a message and what to do with received messages. Such logic may be either proactive or reactive, forming the respective architectural components framed in dashed red lines. The internal logic would typically rely on private internal state, e.g., as encoded in internal databases.

**Table 1:** The Mandrake API for programming agents

Function	Purpose
adapter($\langle \texttt {role}\rangle$, $\langle \texttt {protocol}\rangle$)	Initialize an adapter with a protocol specification and role, so it can check incoming and outgoing messages according to that role’s perspective
adapter.register_reactor($\langle \texttt {schema}\rangle$,$\langle \texttt {handler}\rangle$)	Register a function as a reactor for a schema; whenever that schema is observed, handler is invoked
@adapter.reaction($\langle \texttt {schema}\rangle$)	Decorator syntax for registering a function definition as a reactor
$\langle \texttt {schema}\rangle$($\langle \texttt {parameter}\rangle$...)	Construct a message instance according to a schema from the protocol, using keyword arguments to bind parameters
$\langle \texttt {schema}\rangle$.match($\langle \texttt {parameter}\rangle$...)	Get all enabled instances for a message that match the provided parameters, which are usually usually keys
$\langle \texttt {instance}\rangle$.bind($\langle \texttt {parameter}\rangle$=$\langle \texttt {value}\rangle$...)	Bind multiple parameters at once; an individual parameter can be bound via property assignment
adapter.send($\langle \texttt {instance}\rangle$)	Send a message instance to the recipient specified in the protocol
adapter.start($\langle \texttt {task}\rangle$...)	Start the adapter, listening for incoming messages and running asynchronous tasks such as proactors

The Adapter serves as the interface through which each agent interacts with other agents, enforcing compliance by checking incoming and outgoing messages against what the agent knows about the protocol and previously observed messages. The Adapter consists of the following components:

- Local State, which is responsible for storing the interaction history as observed by the agent.
- Checker, which processes incoming and outgoing messages to update the Local State after verifying that they satisfy protocol constraints.
- Emitter and Receiver, which handle the physical transmission of messages, forwarding parsed messages to the Checker.

The implementation and configuration are up to the system designers, but could be made using components from a standard library such as our reference implementation.

The remaining components constitute the agent internals:

- Internal State, independent of the agent’s interactions
- Policy components: Proactors and Reactors, which emit messages based on internal triggers (such as a schedule) and message reception events respectively. These components can access but not modify the Local State.

We have developed a reference implementation of Mandrake in Python, which is available at https://gitlab.com/masr/mandrake. Our reference implementation realizes the programming model primarily as API functions provided by an adapter library. These API functions, listed in Table 1, are all that is required to implement an agent.

Our example listings are also written in Python using the reference API, and many of them are taken from the example treatment scenario implementation in the above repository.

### Reactors

Reactors are policy components that are registered with the Checker and invoked by the Checker in response to protocol events such as sending or receiving a message. 
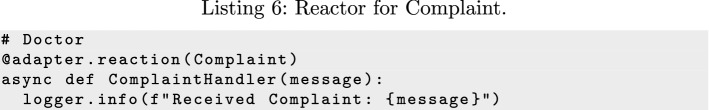


Listing 6 shows a reactor declaration for the *Complaint* message in the Patient agent. Potentially, the role skeleton (describing its reactors) could be automatically generated by tooling from a protocol specification. 
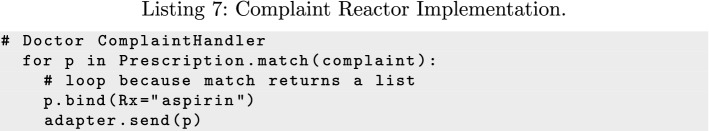


Listing 7 shows a possible complaint reactor implementation, that gets an instance of the *Prescription* message corresponding to the same enactment, binds its Rx parameter to aspirin, and sends it. Requesting a message instance from the enactment automatically fills the known $\ulcorner \mathsf {in}\urcorner$ parameters; in this case ID and symptom.

### Proactors

Proactors are policy components that are not invoked by the Checker; they could be run according to a schedule or some other internal trigger. Proactive policies are necessary for initiating an enactment, because there are no prior events to react to, but other messages in a protocol may also be sent proactively.

For example, to generate the initial *Complaint* in the *Treatment* protocol, Patient could use the following proactive policy function: 
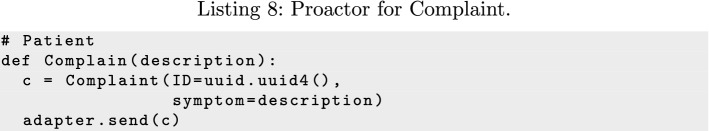


The Complain function is not automatically invoked in reaction to an event, but must be proactively invoked by the agent to initiate the interaction. Perhaps Patient invokes Complain through a suitable user interface, such as a mobile app. Within Complain, a *Complaint* message is constructed, passing in the ID and symptom. Because a *Complaint* message initiates a new enactment, it can be constructed directly from parameters instead of derived from previous observations. Finally, the adapter sends the message.

### Utilizing transformations via agent policies

Once a protocol has been extended either manually or via transformations to support information forwarding and thus fault recovery, the agents need policies to send the messages at the right times. 
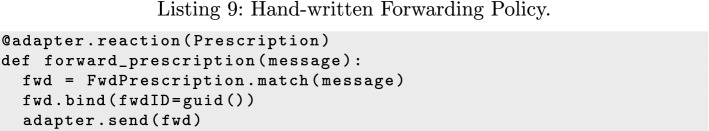


Listing 9 implements a simple reactor in the Doctor for forwarding the *Prescription* message. When Doctor sends *Prescription*, a copy is made with an additional fwdID key, and forwarded to Patient.

Reminder policies are more complicated and challenging to write by hand, since reminders are normally only sent for known messages that have not yet been responded to, often according to a schedule. 
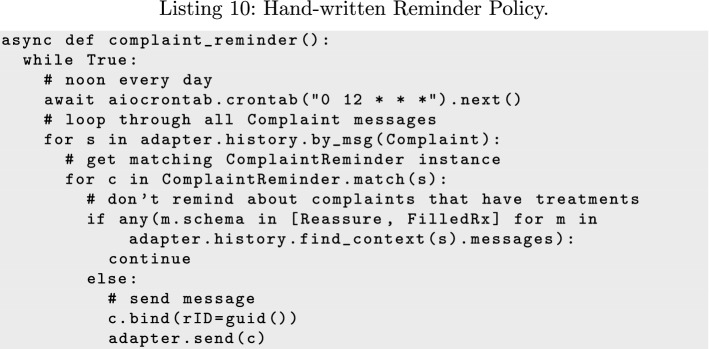


Listing 10 shows how a reminder policy might be implemented in the Patient. The function loops indefinitely, but sleeps until noon each day. At the appropriate time, it scans the history for all sent *Complaint* messages, and sends a reminder for those that do not have a treatment, as evidenced by the observation of a Reassurance or FilledRx message.

Other policies, such as those for the @forward, @route, and @gossip transformations, can be implemented similarly. Fundamentally, every policy either reacts to a message reception (and can use the relevant enactment history) or proactively generates messages according to a schedule.

### Declarative policy specification

Because some fault tolerance policies are common and potentially complex to write directly in low-level proactor and reactor code (e.g., sending reminders, as illustrated in Listing 10), we have implemented a declarative domain-specific language for specifying a class of fault tolerance policies. Instead of implementing the corresponding proactors and reactors, developers can instead specify rules in the language.

#### Syntax

In the syntax description below,    indicates a line break, and brackets indicate optionality. Monospace tokens and quotations are parsed literally, and italic *Tokens* are terms. $+$ indicates the object should be matched one or more times.

$$\mathcal {L}_1$$
Each agent is given a list of policies, with each entry at the same indentation level prefixed by a hyphen and space
$$\mathcal {L}_2$$
Each policy consists of several clauses: an ’action’ clause, and additional condition clauses. The clauses are given by a keyword followed by a colon and space (‘: ’), and then the body of the clause, usually one clause per line.
$$\mathcal {L}_3$$
The body of an action clause itself consists of parts: an action to perform, a list of messages, and optional destination, delay, and preposition subclauses.
  $$\begin{aligned} \textit{Action} \longrightarrow \,&(remind|forward|broadcast)\\&[\texttt {after} \, n \, \texttt {seconds}]\\&[\texttt {until|upon} \, \textit{events}] \end{aligned}$$
$$\mathcal {L}_4$$
The various actions have slightly different structures
  $$\begin{aligned} \textit{remind} \longrightarrow \,&\texttt {remind} \, role \, \texttt {of} \, messages \\ \textit{forward} \longrightarrow \,&\texttt {forward} \, messages \, \texttt {to} \, role \\ \textit{broadcast} \longrightarrow \,&\texttt {broadcast} \, messages \end{aligned}$$
$$\mathcal {L}_5$$
The event subclause of an action identifies an event to react to or wait for
  $$\begin{aligned} \textit{events}&\longrightarrow \, \textit{event}\,((\texttt {or|and})\, \textit{event})+ \\ \textit{event}&\longrightarrow \, (\texttt {received|duplicate}) \, \textit{messages} \end{aligned}$$
$$\mathcal {L}_6$$
A *When* clause describes the schedule for invoking a proactive policy, in traditional cron notation or some frequency in seconds; this clause is left off for reactive policies.
  $$\begin{aligned} \textit{When} \longrightarrow \, \textit{cron string} \,|\, (\texttt {every} \,n\, \texttt {seconds}) \end{aligned}$$
$$\mathcal {L}_7$$
A cron string specifies a period using 5 fields: minute, hour, day, month, weekday. Each field is either a number, or an asterisk (*), meaning ‘every’. Field may also be restricted with $*/n$, meaning ‘every *n*th’. Multiple values can be provided for each field, separated by commas.
  For example, ‘30 12 */2 * *’ means ‘every other day at 12:30’.

#### Examples

Listing 11 shows a policy specification for Patient to remind Doctor about the complaint by forwarding Doctor a new copy of the message on the first of each month. When loaded into Patient’s adapter, this policy specification creates a new Scheduler set to run every day, and registers a generated handler that sends the appropriate reminder. 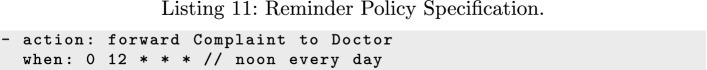


The agent implementation needs to know which message to use for sending a reminder or forward. To avoid coupling the agent implementations, we use an explicit, declarative mapping specification instead of an implicit naming convention: 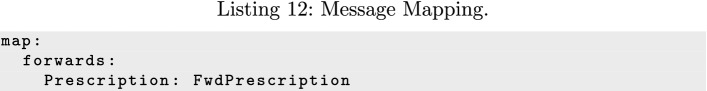


These mapping relationships can be automatically generated by tooling as part of the protocol transformation process.

Besides scheduled actions, the language can specify reactions, such as automatically forwarding information immediately when it becomes available: 
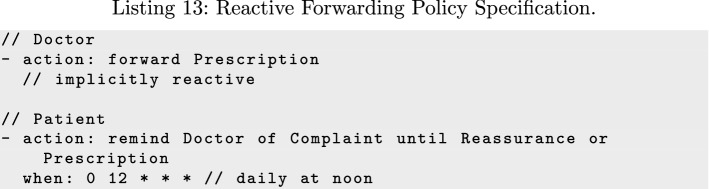


The reactive policy in Listing 13 automatically forwards the *Prescription* message to Patient when it is observed; that is, right after sending it to Pharmacist. The policy registers a reactor for the *Prescription* message that generates a corresponding *FwdPrescription* according to the map and sends it.

The second specification uses the keyword remind as an alias for the generic forward action, and generates two policy components for Patient: a proactor for reminding Doctor of the complaint, and a reactor for *Prescription* and *Reassurance* that deactivates the reminder for the corresponding complaint after receiving one of those two messages. Thus, this policy will stop sending Doctor reminders after the expectation for a treatment is fulfilled, where Listing 11 would continue indefinitely.

These simple policies can be used to construct flexible fault recovery strategies by sending reminders until receiving evidence of progress. For simplicity, forwarding sends the message to all potential recipients by default if no recipients are explicitly specified. Similarly, if a policy does not explicitly specify a condition, it implicitly reacts to the concerned messages. For example, forward Prescription automatically forwards *Prescription* (e.g., by sending *FwdPrescription*) to all potential recipients immediately after *Prescription* is observed (sent). 
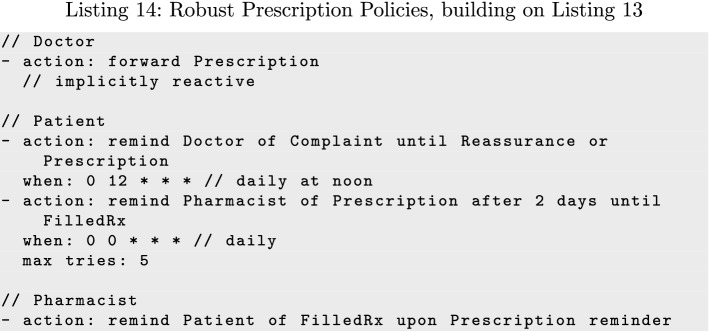


Listing 14 shows how remind and forward can be combined to enable more effective recovery, adding new policies for Patient and Pharmacist to those in Listing 13. Because Patient is the stakeholder desiring FilledRx, it is the best party to judge whether the task has been completed. Forwarding *Prescription* to Patient gives Patient direct recourse to address a delayed or lost prescription by reminding Pharmacist directly. If *Prescription* is lost, the reminder gives Pharmacist the information necessary to send *FilledRx*. If FilledRx is lost instead, the reminder prompts Pharmacist to resend it.

## Evaluation

We evaluate our work conceptually, by explaining how our approach supports application meaning; comparatively, by contrasting it with what would be possible in another MAS framework; and experimentally, by demonstrating how application-level fault tolerance can be effective, practical approach.

### Conceptual evaluation

Our primary contribution is an interaction-oriented programming model designed to support application-level fault tolerance, whose primary competitors are infrastructure-level techniques and ad hoc solutions. Although performance comparisons could be made between our work and examples of competing approaches, such a comparison would at best risk comparing poor versions of each, but would also make a category error. As such, we further develop our argument here regarding the necessity of application meaning and interaction specifications, and show the necessity for an approach like Mandrake.

#### Application meaning

To understand our contribution, it helps to take a quick look at the history of networked applications. The creators of the IP standard, for example, recognized the criticality of application meaning [26]. They observed, correctly, that application meaning varies across applications. Thus, it appeared to them that there would be no systematic way to support application meaning through network abstractions. However, they also saw the need for abstractions. Accordingly, they chose to provide a connection abstraction with reliability and ordering. TCP realizes this abstraction. It supports client-server programming, which relieves programmers of having to model complex interactions and creates the illusion of a distributed finite state machine. The solution was known to be suboptimal even at that time because it violates the end-to-end principle that some of the same researchers had recognized and articulated [47]. In particular, client-server programming is ill-suited to decentralization because it is at odds with the idea of the endpoints carrying out their respective computations largely independently of one another with coupling only where necessary.

In a nutshell, then, computing has faced the dilemma of a design choice between (a) accommodating application meaning but at the cost of working directly on low-level abstractions and (b) using programming abstractions that hide network behavior but at the cost of a more restrictive communication layer that largely subverts decentralization.

We respond to the above dilemma by adopting the abstraction of an *information protocol*. A protocol captures the part of an application’s meaning concerned with how its endpoints interact, leaving their internal details of each endpoint completely hidden. Moreover, an information protocol captures only the essential causal and integrity constraints to achieve a successful interaction—i.e., judged successful according to application meaning. By avoiding spurious constraints, a protocol provides the most flexible computations with respect to application meaning. An abstraction based on protocols is thus both generic and avoids spurious restrictions.

#### Potential causality

Research on implementations based on the dominant paradigm of *potential* causality [40] seek to provide even stronger communication guarantees, such as the ordering observed by distinct endpoints being totally ordered [13]. However, potential causality imagines internal causal connections in an application: a message being sent is assumed to be causally subsequent to any previously received message. Thus, it inherently overestimates the causal relationships. Overestimating the casual relationships leads to well-known problems as brought forth by Cheriton and Skeen [19]; Birman originally resisted this point [12] but has recently acknowledged it in essence [11].

In contrast, information protocols as adopted here capture the *true* causality for each application, though abstracted to the causal connections inferred from interactions without regard to internal computations. In this way, information protocols take the opposite stance on causality and avoid the problems of potential causality.

### Comparison: Jason

A significant part of Mandrake is the programming model, which is intended to simplify agent development, with a special focus on supporting application-level fault tolerance. To evaluate the programming model, we compare it to Jason [17], the agent programming portion of the JaCaMo framework [15]. We compare our work with Jason for these reasons: to show that a popular existing approach does not support application-level fault tolerance (novelty), yet would benefit (significance); and to describe how our work might be applied to other platforms than our own reference implementation (generality).

Jason is an implementation and extension of the AgentSpeak [16] programming language that provides logic programming techniques for building agents according to the Belief-Desire-Intention (BDI) model [51]. Mandrake’s programming model focuses on supporting the interactive aspects of an agent, leaving the internal decision-making to be plugged in; a BDI model could be used to provide decision-making for a Mandrake agent, perhaps using the python-agentspeak library. We have not implemented a BDI reasoner for any Mandrake agents, but parameters learned through message observations match well with terms in a belief base; and Mandrake’s support for both proactive and reactive logic should be able to accommodate any reasoner.

#### Model

Jason is a high-level system for agent development, organizing actions into plans and goals, and using communication abstractions to directly exchange beliefs with other agents and select an action based on the current beliefs. Since communication is abstract, multiple infrastructures can be used for communication, such as a centralized mode for local development, or the JADE agent development framework. JADE in turn can be configured to use multiple transport protocols for sending messages, including HTTP, XMPP[32], and JMS[27]. Each of these protocols aims to provide reliable communication at the infrastructure level; HTTP for instance is based on TCP, which resends packets until they are acknowledged. This approach is effective at dealing with uncommon short-term and seemingly random packet loss.

Protocols are not a first-class abstraction in Jason; instead, communication patterns are coded as part of the agent logic, coupling the agent implementations. Nor are protocols often used as an external specification to guide development, so interaction correctness cannot be verified.

However, Jason’s declarative logic programming style is compatible with an information-based implementation, and provides flexibility that can help with asynchronous messages. If they are properly used, Jason’s rule-based intentions can match the corresponding events regardless of the order they occur, and multiple permutations can be handled with only a few lines.

#### Fault tolerance

Neither Jason nor JADE has specific support for application-level fault tolerance. As such, any fault tolerance will be ad hoc and must be replicated for each message. The code to implement timeouts and retries can easily begin to outweigh the primary interaction logic. However, even if fault tolerance is not supported by the language, the concept of application-level fault tolerance is still important, and a variant of the Mandrake methodology can still be useful.

Plans should be written to progress according to available knowledge instead of simple sequences. For example, instead of sequentially handling the steps of a protocol as in the Contract Net example included with Jason, make each step a separate intention triggered by a belief, such as the arrival of all bids. This decouples the steps and makes it easier to enter them from multiple paths, such as a recovery path, by simply setting the correct beliefs. Then plans should be augmented to track expectations, triggering a recovery plan if the expectation is not met.

This methodology should enable application-level fault tolerance, even if the implementation is manual and tedious. Jason’s support for Java extensions could enable the implementation of a library for Mandrake similar to our Python version.

### Experimental results

We have implemented the system, and performed lightweight experimental evaluations to demonstrate its existence, and possible strengths and weaknesses of the proposed recovery patterns. These experiments were performed using a Linux VM on a laptop, and are not expected to demonstrate best-in-class performance.

The experiment features two parameters: the recovery policies, and the loss rate. The results are displayed in multiple subfigures grouped by the statistic being analyzed: the total enactments completed within the 30 second timeout, the number of messages emitted by Patient, the number of packets sent by Patient, and the rate at which each policy completed its enactments.

The policies examined are (1) a simple reminder policy (referred to as Retry), where Patient reminds Doctor of *Complaint* if their expectation for *FilledRx* is unmet, and Doctor and Pharmacist correspondingly resend any matching messages when reminded to do so, and (2) a checkpoint policy. For the checkpoint policy, Patient reminds Doctor of their complaint until they receive either a copy of their prescription or its fulfillment. Once Patient has received a copy of the prescription, it no longer reminds Doctor but forwards the prescription directly to Pharmacist; the idea being that if the Doctor is busy or unreliable, sending directly will be faster. Once Patient has forwarded the prescription to Pharmacist, they continue reminding Pharmacist of the prescription until it is filled.

These two policies represent conservative and augmented information flows, respectively. The conservative approach, implemented as a simple reminder policy, only sends information along the original pathways provided by the protocol; e.g. the patient only interacts with the doctor. The conservative retry policy is still an application-level policy, but it is somewhat restricted. The checkpoint policy is more proactive, augmenting the protocol with alternative information flows: the patient can now communicate with the pharmacist directly. One objective of this experiment is to show that when implemented correctly, the augmented flows can be at least as good as a basic reminder flow.

The loss rate is the probability that any message sent by a given agent is lost. We simulated losses of 1%, 5%, and 25% (for a consistent factor of 5 between tests), to see how the statistics scale under simulated conditions representing UDP packet loss during transmission, or overwhelmed agents. UDP loss in a network rarely more than a few percent, but more extreme loss rates were included to make the effects of greater loss more apparent.^2^ Instead of simulating all combinations of agent loss rates, we focused on several cases: no loss, one lossy agent with the others unaffected, both non-Patient agents being lossy (since it’s the one doing the recovery) and all emissions equally lossy. Each of these cases is represented by a separate subfigure, with the label indicating which agents are lossy for that expe.riment. During a given step, all lossy agents are set to have the same loss rate.^3^

The experiment was run for five iterations for each combination of loss configuration and recovery policy, to average the results. The sample standard deviations were calculated and included in the graphed data, but are too small to see. The Patient was configured to send 1000 complaints, and expect responses within 1 second (that is, run its recovery policies every second, and wait at least one second before resending a message). Clearly unrealistic for even the most impatient hypochondriac, but still useful as a simulation because our focus is on message loss and recovery. An iteration of the experiment would end as soon as all the enactments were complete, or it had no recovery policy and had not completed any enactments for more than one second. There was also a 30 second timeout that was rarely reached.

**Fig. 2:**
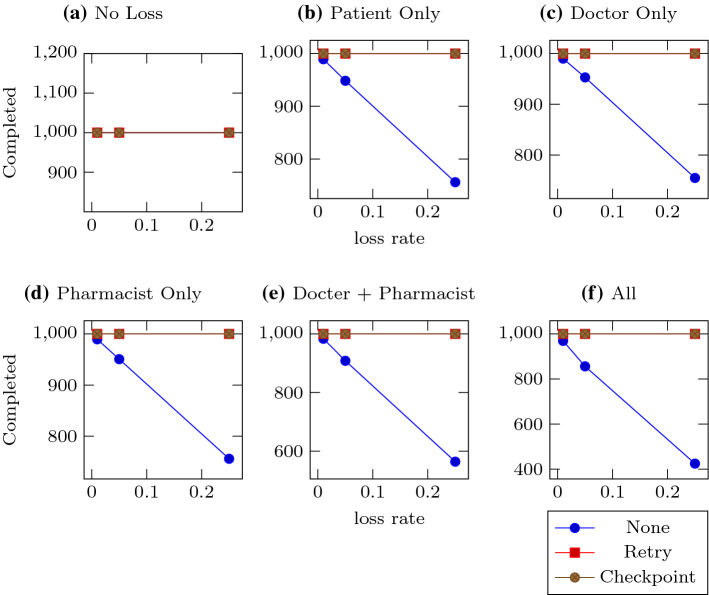
Total enactments completed. Each subfigure represents a different loss configuration, with the lines representing the udieecovery policies. In this figure, a line is also given for the absence of a recovery policy, to show the cumulative effect of the loss rate. Subfigure **a** has no loss, **b**–**d** have one lossy agent, **e** has both doctor and pharmacist lossy, and in **f** all agents are equally lossy. The Y-axes show the number of enactments completed by the timeout, which is set to be 30 seconds. The X-axes are the three different loss rates tested: 0.01, 0.05, and 0.25

Figure 2 shows the total number of enactments completed by each policy for each loss configuration. Both recovery policies were effective at recovering from loss in all cases shown here. We include the results for the cases where the patient does not employ a retry policy to show that completion decreases proportionally to cumulative loss, as expected.

**Fig. 3:**
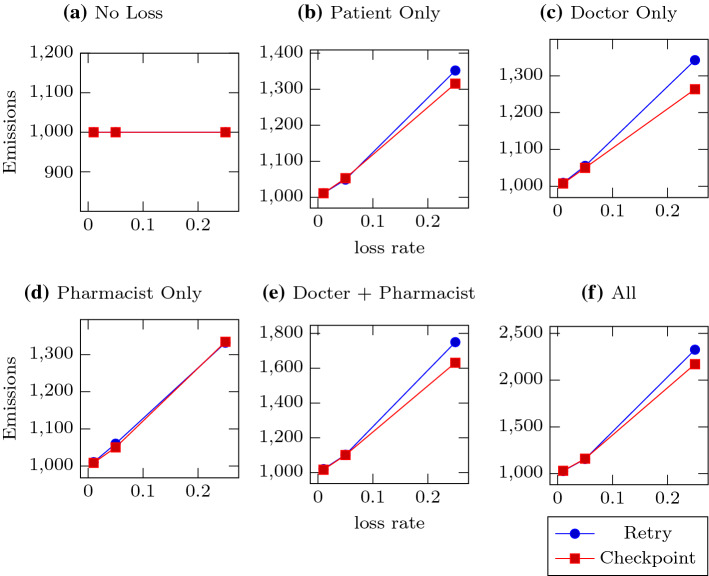
Messages emitted. Each subfigure represents a different loss configuration, with the lines representing the two recovery policies. Subfigure **a** Has no loss, **b**–**d** Have one lossy agent, **e** Has both doctor and pharmacist lossy, and in **f** All agents are equally lossy. The Y-axes show the number of messages emitted by Patient. The X-axes are the three different loss rates tested: 0.01, 0.05, and 0.25

Figure 3 shows the number of message emissions Patient made for each policy and loss configuration. Without any loss, exactly 1,000 messages are emitted. Here we see a slight difference in performance between the two recovery policies. Both have to send additional messages to recover from loss, but the basic retry policy is sensitive to the loss rates of both Doctor and Pharmacist, whereas the checkpoint policy is much less sensitive to Doctor’s loss rate. The checkpoint policy never performs worse than the retry policy, and outperforms it to the greatest extent when only Doctor is lossy. This is because Doctor will sometimes succeed in sending a copy of the prescription to Patient even when failing to send it to Pharmacist, but during the subsequent recovery the patient can send the message to Pharmacist directly via a much more reliable connection.

Note that there is additional overhead incurred by the checkpoint approach; namely that Doctor must send extra copies of the prescription to Patient, doubling the number of messages Doctor must send under normal conditions (in failure conditions some of the reminder work is taken over by Patient). Thus, checkpoint-style policies are likely best used in applications where replicating the information to multiple agents is either already performed as part of the existing requirements (such as monitoring), or of negligible marginal cost.

**Fig. 4:**
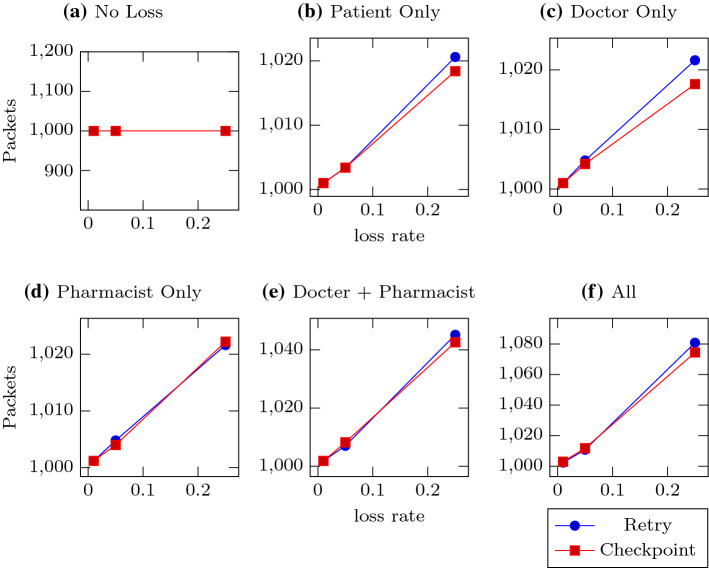
Total packets sent. Each subfigure represents a different loss configuration, with the lines representing the two recovery policies. Subfigure **a** Has no loss, **b**–**d** Have one lossy agent, **e** Has both doctor and pharmacist lossy, and in **f** All agents are equally lossy. The Y-axes show the number of UDP packets sent by Patient. The X-axes are the three different loss rates tested: 0.01, 0.05, and 0.25

Figure 4 shows similarly shaped results for the number of packets that Patient emits, though the total numbers are much lower. This is because the policies are batch processes, and may find multiple enactments that require action (resending, forwarding, etc.) at the same time; when multiple messages are generated at the same time, our implementation groups the messages by recipient and encodes them into as few packets as it can. When the messages are small, as in this experiment, the savings can be significant—here, the number of messages ranged from 1000 to  3000, but the number of packets only ranged from  1000 to  1080.

**Fig. 5:**
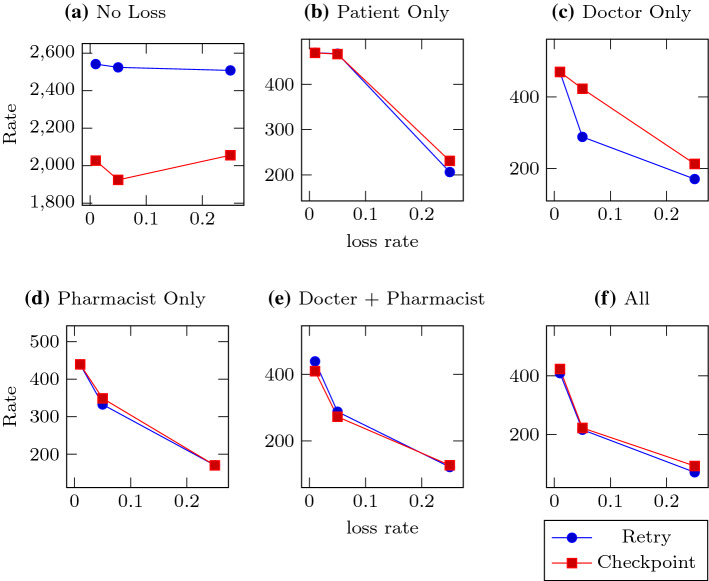
Rate of completion (enactments/second). Each subfigure represents a different loss configuration, with the lines representing the two recovery policies. Subfigure **a** has no loss, **b**–**d** have one lossy agent, **e** has both doctor and pharmacist lossy, and in **f** all agents are equally lossy. The Y-axes show the number of enactments completed (by observing *FilledRx* or one of its reminders) divided by the duration of the iteration. The X-axes are the three different loss rates tested: 0.01, 0.05, and 0.25

The final graph, Fig. 5, shows the rate at which each policy completed enactments, in enactments per second. The first configuration, where none of the agents are lossy, shows that the simple retry policy is slightly more efficient to execute than the checkpoint policy. In all other cases, their performance seems similar, with the only notable difference being again the Doctor-only case, where the checkpoint policy’s throughput is much less effected by loss than the retry policy.

## Related work

Fault handling may be thought as a kind of exception handling. However, exception in the context of programming languages, including process languages such as BPMN [42], are handled by a centralized language runtime. Plus, exceptions and exception handling are implemented via out of band signaling, which adds complexity to the program. By contrast, Mandrake addresses faults in decentralized MAS, specifically, faults understood as the violation of expectations in the enactment of protocols. Agents with expectations may initiate recovery, in alignment with the protocol. Fault tolerance in Mandrake merely involves the addition of alternative paths to completion, such as retries and forwarding.

Software systems built using the actor model, where processes are encapsulated in actors that interact with their environment through simple messages, have long been used to build fault-tolerant distributed systems [5]. The actor model [2, 3] is conducive to fault tolerance both because the simple messaging interface of actors minimizes coupling and enables testing, and because actors easily model physical distribution, which is necessary for eliminating single points of failure.

Actor-based systems commonly address fault tolerance through supervision hierarchies. If an actor throws an error, its supervisor receives the error and can respond by restarting the actor, killing dependent actors, or possibly dying and throwing an error itself. These supervision hierarchies enable the developer to design fault tolerance at the application level, and can be supported by libraries such as Erlang’s OTP [18] to minimize development effort.^4^ However, Erlang does not have specific support for programming application level message retries, relying on TCP for automatic packet-level retries and supervisor-triggered restarts when the connection breaks. While supervision could conceptually be used to implement application-level retries, supervisor hierarchies in Erlang/OTP are specifically for detecting process failures, and restarting failed processes and any processes that depend on them. This mechanism for restarting crashed agents is complementary with Mandrake, which focuses on recovering lost information between live agents.^5^ Actor system fault tolerance also usually focuses on handling signaled errors—errors that do not immediately crash the actor, but are instead reported via some status message for another actor to handle. Such signals are helpful for handling domain-specific problems that prevent progress (Mandrake’s retries will not help if the Pharmacist is waiting for a delivery from their supplier), or which need to be resolved by another party (perhaps the Pharmacist needs to notify their supplier when they run out of stock).

To avoid hidden coupling, all communications between agents should be explicitly specified; thus the status and error messages should be added to the protocol specification. Signaled errors are thus compatible with Mandrake; we merely focused on the aspects that Mandrake specifically addresses. However, signaled errors cannot handle cases where information was lost (e.g. the Pharmacist could not signal an error for a prescription it never received), or when an unexpected crash occurs (e.g. a hardware failure, preventing any signals from being sent); Mandrake provides a solution for these situations through expectations. Finally, most actor systems rely on infrastructure for reliable messaging and process messages sequentially, expecting FIFO ordering. Relying on FIFO enables the developers to avoid explicitly modeling actor state, and TCP signals when a connection breaks simplifying supervision. In contrast, Mandrake’s explicit information model relieves the dependence on FIFO for state alignment, and uses expectations to detect agent failures so communication can survive temporary network disruptions.

Microservices are an increasingly common architecture and means of deployment. Each microservice is intended to encapsulate a single application concern, exposed as a web service for easy composition, and deployed in an isolated environment to reduce coupling and interference from other components [41]. Traditional web-based interactions are asymmetric, with the client merely reflecting state held by the server. However, microservices are often clients themselves, and depend on other services to produce their outputs, exposing them to communication faults. To some extent fault tolerance is transparently added to microservices via *sidecars* or *service meshes* such as Istio [34], proxies that manage requests and retries on behalf of the microservice. In contrast, Mandrake assumes a decentralized system where each agent manages its own state and messages, enabling more flexible and robust communication patterns between agents.

Fisher et al. [31] discuss a framework for certifying reliable autonomous entities, but consider the problem at the social and regulatory levels. Such an approach is useful for identifying the methods and processes can be used to certify that an agent will behave as it ought despite its autonomy, but does not support designing a system that can complete objectives despite faults.

Much work has been done to support programming models for agent development, such as the JaCaMo framework and extensions thereto [7, 9, 45, 46] and JADE [10]. However, most existing work on programming models is focused on implementing the agent logic rather than supporting interactions, and little effort has focused on supporting fault tolerance. IODA is an approach for multi-agent simulations that does have a model of interactions [38], though it does not specifically support application-level fault tolerance.

There is MAS work focused on exception handling in multiagent contexts: Klein and Dellarocas [37] propose a shared exception handling service that other agents turn to for help recovering from a problem, such as when a plan fails or a garbled message is received. Platon et al. [44] surveyed the challenges for exception handling in MAS, and Platon’s thesis is on exception handling and a robust framework for executing agent plans [44]. Lam et al. [39] describe a workflow management system that uses norms and semantic web techniques to handle exceptions that arise during enactment. Mandrake takes a more agent-local and interaction-oriented approach, with each agent responding to any violations of its own expectations using communication patterns supported by an interaction protocol.

Outside of programming models and exception handling, there is also much work on protocols, modeling, verification, and monitoring of MAS behavior.

Several approaches model protocols by specifying an ordering of messages via some control-flow expression, e.g., via a state machine or trace expression. Baldoni et al. [8] present a state machine-based view of interaction from the agent standpoint and show how interoperability of agents can be ensured provided they conform with the endpoint specification based on the role they adopt in a protocol, the main challenge there being to guarantee that the agents don’t make incompatible choices. Ferrando et al. [30] specify protocols as trace expressions, with recent work on enactability. Winikoff et al. [57] proposes a graphical protocol notation based on hierarchical state machines combined with logical predicates for improved usability and expressiveness without sacrificing formal verification.

The control flow-based languages typically assume reliable, FIFO delivery of messages between pairs of agents. However, as we saw earlier, ordering assumptions interfere with agent autonomy by limiting the choices available to them. And reliability assumptions are generally insufficient for purposes of fault tolerance since what counts as a fault and recovery are application-level considerations. One could argue that by specifying message ordering, the control flow-based languages lift the ills of ordering to the application level.

By contrast, BSPL, by specifying information causality and integrity, enables implementing protocols with unordered, unreliable communication. Not relying on ordering in fact helps to easily implement fault tolerance at the application level. For example, to handle message loss, agents have the luxury of being able to retry a transmission whenever they want. And agents can receive such a retransmission at any time. Further, receiving a duplicate has no effect in BSPL because it simply brings already known information.

Dastani et al. [28] present an approach on monitoring agent behavior against specified norms. Günay et al. [33] study commitment protocols, and how to dynamically adapt them to changes in the environment or in the agents to ensure goals could still be reached. Baldoni et al. [7] use type checking for roles in a commitment protocol. In the information protocol framework underlying Mandrake, the compatibility of the choices allowed to agents (via roles) by a protocol are checked through verifying safety and liveness of a protocol [53, 55]. The Mandrake adapter uses runtime protocol monitoring to ensure compliance and support fault recovery policies.

Agent-based approaches are now being employed for applications such as the Internet of Things [25] to improve flexibility (because agents can use multiple means to achieve a goal) and responsiveness (because agents act independently without relying on orchestration) in such a decentralized setting. These objectives would benefit from an approach such as ours, and show how our work could be useful for existing web and IoT applications.

Today, most developers rely on existing protocols such as TCP for communication between services or agents. A few build a new infrastructure-level protocol for their needs that may later become standardized, such as uTP [14] and QUIC [35]. Rarely is application-level information used to inform network-level fault recovery; the closest examples might be job-queuing systems that distinguish between message reception and job completion.

Many network protocols such as TCP use acknowledgments to confirm packet delivery, and thus quickly detect and recover from packet loss [4]. Acknowledgments can also be used at the application level, but we do not focus on application-level acknowledgments because their similarity to packet acknowledgments distracts from the broader significance of application-level fault tolerance; we discuss them briefly here instead. Network-level acknowledgments are not meaningful: they only confirm packet delivery, not whether the information has been received or processed by the application. Conversely, application-level acknowledgments are less efficient for large data transfers because decision-making and autonomy, necessary for meaning, increase latency and overhead. However, application-level acknowledgments convey the meaning that the information was observed and acknowledged by the agent; they provide a basis for commitments, and indicate that the information is being processed—likely an acknowledged message has been logged, and could be restored even if the agent crashes.

## Discussion: conclusions and future directions

In this paper we have raised the topic of meaning-based application-level fault tolerance. Every application has its own objectives and criteria for success, and fault tolerance must work toward those goals, not just ensure individual steps. Accomplishing application objectives requires awareness and utilization of application meaning; infrastructure-level techniques do not have access to application-level information and so cannot solve application-level problems. Infrastructure-level techniques are used because they are well understood, optimized, and generic—any problem that uses the infrastructure can theoretically benefit from them. To close this gap between easy-to-use infrastructure-level techniques and the application-level solutions we need, specifically in the MAS domain, we propose an interaction-oriented agent programming model with first-class support for fault tolerance. However, our work is just the beginning. We now discuss several possible directions for future work:

*Multicast errors* Although many common system designs use a pairwise interaction model compatible with our current work, there are other communication patterns for which the compatibility is less clear. One important direction for extending our work might be to determine Mandrake’s limits for handling multicast communication and then address any revealed problem, possibly by extending our framework to include patterns for consensus protocols or other aggregation.

*Role replacement* One possible way to restore progress in a decentralized application despite unavailability is to identify new business partners. That is, the agents enacting a protocol could give up on a counterparty that is nonresponsive, and find another agent to fill the role. For example, Patient could choose a different Doctor or Pharmacist if Patient is unsatisfied with their service.

*Porting to other platforms* Mandrake is currently implemented for BSPL on a Python-based agent development framework. However, the concepts proposed by Mandrake should be applicable to other agent development frameworks. For example, a Mandrake-style protocol adapter could be implemented for JADE or JaCaMo on top of their existing speech-act communication systems, which would enable the enactment of BSPL protocols on those platforms. Or, a BDI-based system for expectations and recovery policies could be built, integrating Mandrake concepts more natively. It would also be interesting to generate Jason plan skeletons using our declarative policy specifications.

*Dynamically adjusting transmitted information* Although a message’s dependencies may be useful to track for provenance, not all information dependencies are useful for the recipient, especially if they are already known. Redacting unused parameters may save bandwidth and decoding time.

To support such an optimization, we conceptually distinguish between four representations of a message: the schema, instance, bun, and packet. The schema is the abstract specification of a message, and is not associated with a particular payload or enactment. An instance consists of a message schema plus information bindings, and is associated with an enactment. A *bun* is an instance of a message that has been serialized for transmission; it may have more or fewer parameters than the message schema, so long as the recipient can reconstruct the complete instance upon reception. Finally, the packet is the physical message (e.g., UDP) transmitted by the infrastructure, though some transports (such as TCP) may not have application-visible packets. We distinguish between bun and packet because a bun is the representation of a complete message; one bun may be split across multiple packets, or multiple buns may be batched into a single packet.

*Congestion control* Enabling the switch from TCP to UDP may gain in latency and overhead but risks problems such as congestion collapse. Such problems may not be apparent in simple simulations but can make real-life implementations difficult.

By sharing information with the Receiver, the Emitter can be aware of statistics for each channel. By aggregating timestamp observations, the Emitter can estimate delay, which is an indicator for traffic congestion.

Using information about observed delays and retries, a range of congestion policies can be implemented, such as TCP-style backoff, or adjusting transmission rate based on observed delay, as in Low Extra Delay Background Transport (LEDBAT) [49].

In our estimation, based on the end-to-end principle, all decisions that could affect the application should be made at the application level. That does not mean that the developer *must* make such decisions, just that they should be able to. Separating the system into layers makes those decisions inaccessible to higher layers of the system. Thus, we propose and demonstrate an architecture that uses encapsulation instead of layering to hide complexity. The adapter can provide default implementations of e.g., congestion control (possibly identical to TCP), but the developer can choose to override them at any time. In this way, flexibility can be gained without also increasing complexity for the developer.

*Improved programming model* Our current programming model, based on classic Web request handling patterns, is easy to learn and greatly simplifies certain challenges of asynchronous message handling like message correlation via the match API. Unfortunately, reactive handlers for each incoming message burden developers with control flow decisions that should already be covered by the protocol specification; for example, an action that should be taken after two messages are received in any order would need two handlers, one for each message, each with a check for whether the other has been received yet or not. An alternative programming model could focus on the enabled messages, abstracting away the enabling events. The outstanding challenge for developing such a model is how to handle cases where more than one message becomes enabled at the same time, especially if the generators are asynchronous. We are also pursuing an integration of our information protocol approach with Jason BDI agents.

Mandrake is applicable to any MAS since it makes no assumptions regarding the coupling between agents. That is, it addresses the most general setting where the agents are loosely coupled and can behave in any way they like. Given a protocol, Mandrake provides means to have the agents interact in a robust manner. Mandrake does impose some requirements on the interaction, however, through its adoption of BSPL; namely, BSPL requires a key-based information structure and so may not be compatible with existing systems. BSPL is also limited from doing certain kinds of multicast and streaming interactions. In future work Mandrake could be implemented to support BSPL extensions for multicast and streaming interactions.

## Footnotes

1 $$\Leftarrow \mathrm{r3:semantic-identifiers}.$$

2 $$\Leftarrow \mathrm{r3:loss-rate}.$$

3 $$\Leftarrow \mathrm{r3:cases}.$$

4 $$\Leftarrow \mathrm{r2:supervision}.$$

5 $$\Leftarrow \mathrm{r2:signaled-errors}.$$
